# Supplementary material for: Trends in Mental Wellbeing of US Children, 2019–2022: Erosion of Mental Health Continued in 2022
Source: Int J Environ Res Public Health. 2024 Jan 25;21(2):132. doi: 10.3390/ijerph21020132 (PMC10887976; doi:10.3390/ijerph21020132)

Table S1: Correlates of poor mental health outcomes by age group				
Variables	Full Sample	2-5 Years	6-11 Years	12-17 Years
Age	0.05*	0.20*	0.00	-0.30*
Female Sex	-0.32*	-0.25*	-0.53*	-0.17*
Race				
White (Reference)				
Black	-0.43*	-0.08	-0.57*	-0.57*
Hispanic	-0.33*	-0.12*	-0.44*	-0.37*
Asian	-0.64*	-0.18*	-0.71*	-0.90*
American or Indian native	-0.28*	-0.22	-0.42	-0.14
Other	-0.46*	-0.24	-0.51*	-0.53*
Household Size	-0.03	-0.05	-0.09*	-0.02
Good or better self-rated health	-2.60*	-1.22*	-2.80*	-2.78*
Insurance coverage				
Private (Reference)				
Medicaid and Other public	0.24*	0.05	0.23*	0.40*
Other	0.10	0.17	-0.03	0.13
Uninsured	-0.25*	-0.05	-0.33*	-0.24*
Family Structure				
Single Parent (Reference)				
Married two parents	-0.19*	0.03	-0.17	-0.27*
Cohabitating Two parent	-0.24*	-0.03	-0.24	0.16
No parent	0.26	0.28	0.47	0.12
Parental Age (First residential parent)	0.00	0.00	0.01	0.00
Family Race Composition				
Same race family (Reference)				
Mixed race family	0.52*	0.26	0.49*	0.72*
Unknown or no parent	-0.18*	-0.08	-0.17	-0.23*
Parental Employment	-0.18*	-0.03	-0.17*	-0.31*
At least one parent has a college degree	0.00	-0.09	0.05	0.00
Federal Poverty Line based family income categories				
0.0-0.49 *(Reference)*				
0.50-0.74	0.07	0.27	0.06	-0.17
0.74-0.99	-0.03	-0.01	0.18	-0.30
1.00-1.24	0.04	0.27	0.02	-0.18
1.25-1.49	-0.17	-0.03	0.03	-0.56*
1.50-1.74	-0.12	0.01	-0.04	-0.37
1.75-1.99	0.05	0.09	0.23	-0.27
2.00-2.49	-0.15	-0.08	-0.26	-0.27
2.50-2.99	-0.01	-0.03	0.02	-0.13
3.00-3.49	-0.04	-0.01	-0.12	-0.07
3.50-3.99	0.04	0.00	-0.08	0.06
4.00-4.49	0.05	-0.13	0.16	-0.08
4.50-4.99	-0.08	-0.14	-0.19	0.00
5.00 or greater	-0.07	-0.13	-0.15	-0.08
High Food security	-0.50*	-0.14	-0.62*	-0.61*
Owned Residence	-0.06	-0.04	-0.12	0.00
At least one elderly person lives in house	0.15	0.28	0.12	0.11

1. Single asterisk (*) indicates significance at $p<0.05$
2. Coefficients indicate change in summary scores for mental distress. Summary scores were unweighted sums of responses to binary survey items. Summary score maximums varied by age subgroup depending on the total number of survey items asked of respondents belonging to each subgroup

Figure S1: Mean prevalence of key metrics of mental wellbeing across study years in all children by domain

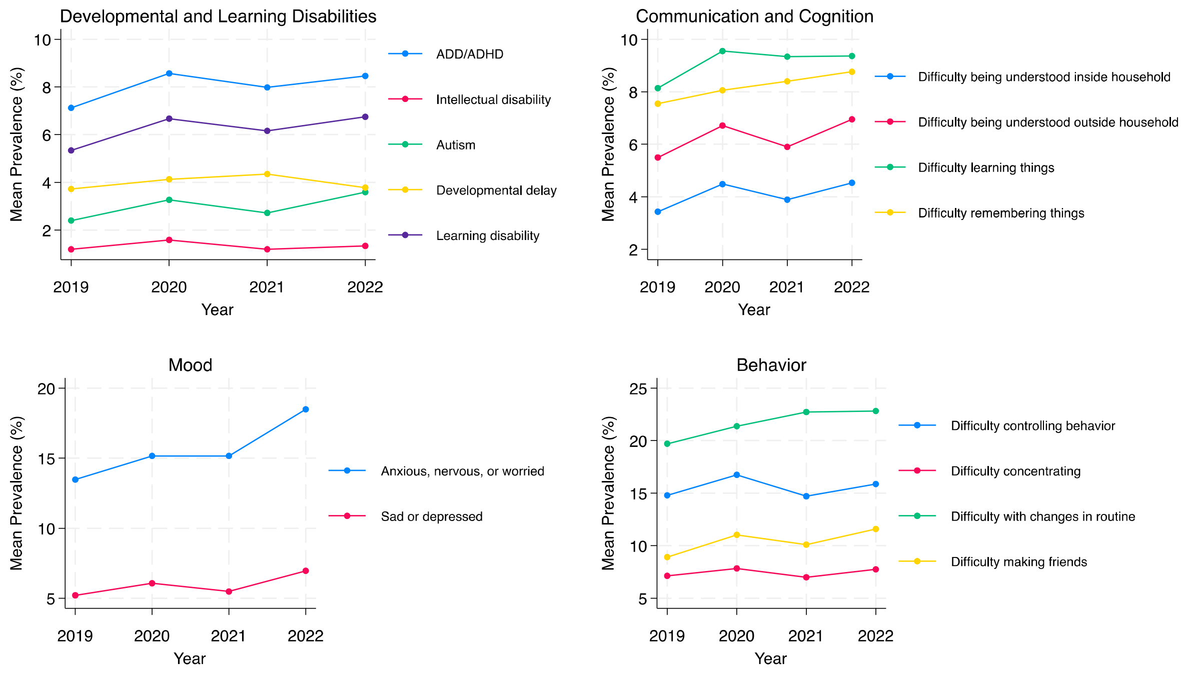

Supplement: Supplementary file 1 [file ijerph-21-00132-s001.zip › ijerph-2818093-supplementary.pdf]
